# Supplementary material for: Cooperative siderophore use stabilizes a protective leaf microbiome
Source: bioRxiv. 2026 Mar 18:2026.03.18.712463. Preprint. [Version 1] doi: 10.64898/2026.03.18.712463 (PMC13015310; doi:10.64898/2026.03.18.712463)
Supplement: 1 [file NIHPP2026.03.18.712463v1-supplement-1.pdf]

bioRxiv preprint doi: https://doi.org/10.64898/2026.03.18.712463; this version posted March 18, 2026. The copyright holder for this preprint (which was not certified by peer review) is the author/funder, who has granted bioRxiv a license to display the preprint in perpetuity. It is made available under aCC-BY 4.0 International license.

Name	scan1	m/z	GNPS_library	scan2	m/z	GNPS_library	EdgeAnnotation
85917 (-) 85951	85917	518.32401	PC(18:3/0:0); [M+H]+ C26H49N1O7P1	85951	286.6249	#N/A	[M+Fe+2]+2 [2M+2H]+2 dm/z=231.69907
85884 (-) 85951	85884	1035.6385	#N/A	85951	286.6249	#N/A	[M+Fe+2]+2 [2M+H]+ dm/z=749.01351
89758 (-) 89842	89758	480.30827	Spectral Match to 1-(9Z-Octadecenoyl)-sn-glycero-3-phosphoethanolamine from NIST14	89842	267.6173	#N/A	[M+Fe+2]+2 [M+H]+ dm/z=212.69094
90415 (-) 90241	90415	288.64064	#N/A	90241	522.3553	Spectral Match to 1-(9Z-Octadecenoyl)-sn-glycero-3-phosphocholine from NIST14	[M+Fe+2]+2 [M+H]+ dm/z=233.71462
104651 (-) 104701	104651	324.28943	#N/A	104701	378.2088	#N/A	[M+Fe+2-H]+ [M+H]+ dm/z=53.91933
10623 (-) 11099	10623	689.34642	#N/A	11099	398.0882	#N/A	[M+Fe+3-2H]+ [2M+H]+ dm/z=291.25819
10848 (-) 11099	10848	345.17671	Rhodotorulic acid	11099	398.0882	#N/A	[M+Fe+3-2H]+ [M+H]+ dm/z=52.91153
32289 (-) 33426	32289	619.36629	Desferrioxamine G	33426	336.6423	#N/A	[M+Fe+3-H]+2 [M+H]+ dm/z=282.72401
36653 (-) 36511	36653	420.04145	#N/A	36511	786.1647	FLAVIN ADENINE DINUCLEOTIDE	[M+Fe+3-H]+2 [M+H]+ dm/z=366.12326
96862 (-) 97137	96862	610.36758	#N/A	97137	279.2317	Spectral Match to 9(10)-EpOME from NIST14	[M+H]+ [2M+Fe+3-2H]+ dm/z=331.13592
9952 (-) 10848	9952	742.25815	#N/A	10848	345.1767	Rhodotorulic acid	[M+H]+ [2M+Fe+3-2H]+ dm/z=397.08144
97068 (-) 96862	97068	261.22113	#N/A	96862	610.3676	#N/A	[M-H2O+H]+ [2M+Fe+3-2H]+ dm/z=349.14645
104502 (-) 104361	104502	325.27343	#N/A	104361	396.1954	#N/A	[M-H2O+H]+ [M+Fe+3-2H]+ dm/z=70.92199
104361 (-) 104523	104361	396.19542	#N/A	104523	342.3	#N/A	[M-H2O+NH4]+ [M+Fe+3-2H]+ dm/z=53.89542

Supplementary Table 1 Summary of the Ion Identity Molecular Network (IIMN) edges, including Fe adducts. Edges connect metabolites (nodes) with iron adducts detected in the native metabolomics experiments in which iron salts were added to the system (Fig. 2F), including the siderophores rhodotorulic acid and desferrioxamine G.

bioRxiv preprint doi: https://doi.org/10.64898/2026.03.18.712463; this version posted March 18, 2026. The copyright holder for this preprint (which was not certified by peer review) is the author/funder, who has granted bioRxiv a license to display the preprint in perpetuity. It is made available under aCC-BY 4.0 International license.

Strain	Total Read Pairs (M)	Total Bases (Gbp)	Total Read Pairs (Trimmed) (M)	Total Bases (Trimmed) (Gbp)	Assembled Size (Mbp)	Num Contigs	N50 (kb)	GTDB Assignment
wgs_S12036Nr1	8.08	2.41	7.94	2.37	6.69	637	105.7	s__Pseudomonas_E koreensis_A
wgs_S12036Nr2	8.10	2.42	7.96	2.37	5.92	229	247.5	s__Pseudomonas_E siliginis
wgs_S12036Nr3	8.10	2.41	7.95	2.37	5.94	281	112.3	s__Pseudomonas_E moraviensis_A
wgs_S12036Nr4	8.07	2.41	7.92	2.37	5.97	244	174.2	s__Pseudomonas_E moraviensis_A
wgs_S12036Nr5	8.08	2.41	7.93	2.38	6.04	207	249.7	s__Pseudomonas_E koreensis_F
wgs_S12036Nr6	8.12	2.42	7.98	2.38	5.92	249	233.7	s__Pseudomonas_E siliginis
wgs_S12036Nr7	8.11	2.42	8.03	2.39	6.03	228	101.7	s__Pseudomonas_E viridiflava
wgs_S12036Nr8	8.04	2.41	7.90	2.36	5.92	241	225.9	s__Pseudomonas_E siliginis
wgs_S12036Nr9	8.10	2.42	7.93	2.37	6.03	289	305.8	s__Pseudomonas_E moraviensis_A
wgs_S12036Nr10	8.11	2.42	7.97	2.38	6.05	249	196.2	s__Pseudomonas_E moraviensis_A
wgs_S12036Nr11	8.11	2.42	7.96	2.38	5.97	282	154.9	s__Pseudomonas_E moraviensis_A
wgs_S12036Nr12	8.06	2.41	7.92	2.36	5.97	278	164.4	s__Pseudomonas_E moraviensis_A

Supplementary Table 2: Whole-genome sequencing of commensal strains used in this study, with associated metadata including isolation site and genome features.
